# Thyroid hormone enhances stem cell maintenance and promotes lineage-specific differentiation in human embryonic stem cells

**DOI:** 10.1186/s13287-022-02799-y

**Published:** 2022-03-21

**Authors:** Chunhao Deng, Zhaoying Zhang, Faxiang Xu, Jiaqi Xu, Zhili Ren, Carlos Godoy-Parejo, Xia Xiao, Weiwei Liu, Zhou Zhou, Guokai Chen

**Affiliations:** 1 Centre of Reproduction, Development and Aging, Faculty of Health Sciences, University of Macau, Macau SAR, China; 2 Institute of Translational Medicine, Faculty of Health Sciences, University of Macau, Macau SAR, China; 3 Bioimaging and Stem Cell Core Facility, Faculty of Health Sciences, University of Macau, Macau SAR, China; 4 State Key Laboratory of Cardiovascular Disease, Beijing Key Laboratory for Molecular Diagnostics of Cardiovascular Diseases, Diagnostic Laboratory Service, Fuwai Hospital, National Center for Cardiovascular Diseases, Chinese Academy of Medical Sciences and Peking Union Medical College, Beijing, 100037 China; 5 MoE Frontiers Science Center for Precision Oncology, University of Macau, Macau SAR, China

**Keywords:** Thyroid Hormone, Triiodothyronine (T3), hPSCs, Pluripotency, Cell culture, Trophoblast

## Abstract

**Background:**

Thyroid hormone triiodothyronine (T3) is essential for embryogenesis and is commonly used during in vitro fertilization to ensure successful implantation. However, the regulatory mechanisms of T3 during early embryogenesis are largely unknown.

**Method:**

To study the impact of T3 on hPSCs, cell survival and growth were evaluated by measurement of cell growth curve, cloning efficiency, survival after passaging, cell apoptosis, and cell cycle status. Pluripotency was evaluated by RT-qPCR, immunostaining and FACS analysis of pluripotency markers. Metabolic status was analyzed using LC–MS/MS and Seahorse XF Cell Mito Stress Test. Global gene expression was analyzed using RNA-seq. To study the impact of T3 on lineage-specific differentiation, cells were subjected to T3 treatment during differentiation, and the outcome was evaluated using RT-qPCR, immunostaining and FACS analysis of lineage-specific markers.

**Results:**

In this report, we use human pluripotent stem cells (hPSCs) to show that T3 is beneficial for stem cell maintenance and promotes trophoblast differentiation. T3 enhances culture consistency by improving cell survival and passaging efficiency. It also modulates cellular metabolism and promotes energy production through oxidative phosphorylation. T3 helps maintain pluripotency by promoting ERK and SMAD2 signaling and reduces FGF2 dependence in chemically defined culture. Under BMP4 induction, T3 significantly enhances trophoblast differentiation.

**Conclusion:**

In summary, our study reveals the impact of T3 on stem cell culture through signal transduction and metabolism and highlights its potential role in improving stem cell applications.

**Supplementary Information:**

The online version contains supplementary material available at 10.1186/s13287-022-02799-y.

## Background

Human pluripotent stem cells (hPSCs), with their potential to generate all the cell types of the human body, hold great promises in regenerative medicine [1]. In order to realize their production and clinical applications, effective cell culture technologies in maintaining pluripotency and inducing specific cell fates are indispensable, and optimized, chemically defined culture platforms must be established [2]. In the past decades, a lot of valuable results have been obtained from stem cells cultured in traditional, serum-containing media, yet some hPSC behavior cannot be recapitulated in defined culture. An examination of potential players contributing to these differences will enable us to identify novel regulators of stem cell pluripotency and differentiation.

One major difference between hPSCs maintained in chemically defined media and those in feeder cell culture is the dependence on high levels of growth factors [3]. Comparing to chemically defined E8 system, much less FGF2 is required to maintain hESC pluripotency in traditional feeder cell culture containing serum, Knockout Serum Replacement (KOSR), or AlbuMAX. hESC maintenance in albumin-free E8 medium requires 100 ng/ml of FGF2 [4]. However, 4 ng/ml FGF2 is sufficient to maintain pluripotency in traditional feeder cell culture containing AlbuMAX [5]. AlbuMAX is a serum albumin extract that potentially carries various bioactive factors including hormones and lipids [6]. It is plausible that some albumin-binding factors could benefit hESC maintenance.

Hormones play key roles in growth and development, but the impacts of hormonal control on stem cell maintenance and differentiation remain largely unexplored. As a central regulator of energy homeostasis, thyroid hormone has been shown to be essential for embryogenesis and fetal development [7–10]. Deficiency of thyroid hormone reduces fertility and decreases implantation efficiency after in vitro fertilization (IVF) [11, 12], implying its importance in early embryogenesis. During the implantation process, trophoblasts secrete human chorionic gonadotropin (hCG) hormone to stimulate maternal thyroid hormone production, which supports fetus development [13, 14]. At the same time, thyroid hormone exerts cell-type specific effect on gene expression in inner cell mass and trophoblasts in blastocyst [15]. Despite the critical roles of thyroid hormone, its molecular mechanisms in embryogenesis are still to be studied in more details.

There are two main forms of thyroid hormone in the human body, triiodothyronine (T3) and thyroxine (T4). T3 is the active form of thyroid hormone, and T4 is converted to T3 to exert its effects. T3 acts mainly through its nuclear receptors TRα and TRβ to modulate transcription related to various critical processes, including metabolism, growth and development [16]. The majority of thyroid hormone in the blood is bound to albumin [17].

In this report, we identified T3 as one of the bioactive components present in fetal bovine serum, KOSR and AlbuMAX, and systemically studied the effect of T3 on hPSC maintenance and differentiation. T3 improves the stability of hPSC culture under suboptimal conditions, significantly decreases the dependence on FGF2 and promotes extraembryonic differentiation. Our findings show that T3 is a potent regulator of hPSC pluripotency and cell fate determination.

## Material and methods

### hPSC culture and maintenance

H1 and H9 hESCs and NL-1 hiPSCs were used in this study. H1 cells were used in most of the work unless specifically described. hPSCs were cultured and maintained in E8 medium as previously described [4]. E8 medium was prepared by combining the following components: 1X DMEM/F12, insulin (10 μg/ml), FGF2 (100 ng/ml), TGFβ (1.74 ng/ml), transferrin (10 μg/ml), ascorbic acid (64 mg/l) and sodium selenite (13.6 μg/l). Cells were passaged every 3–4 days using the EDTA method as previously described [18]. TrypLE Select (Thermo Fisher, 12563029) was used for cell dissociation before counting following manufacturer’s instructions.

### hESC differentiation

Lineage-specific differentiation of hESCs was conducted as previously described [19]. Briefly, hESCs were passaged to 12-well plates at a ratio of 1:12. Differentiation starts on day 2 after passaging for endoderm differentiation and starts on day 1 after passaging for all the other lineages. For trophoblast differentiation, cells were differentiated in E7 medium (E8 medium without FGF2) with 20 ng/ml BMP4 for 6 days with medium change every 2 days and then harvested on day 6 for RT-qPCR analysis of *CGA*, *CGB*, *GCM1*, *GATA2* and *TROP2*. For mesoderm differentiation, cells were differentiated in E8 medium supplemented with 20 ng/ml BMP4 for 2 days and harvested on D2 for RT-qPCR analysis of *TBXT* and *MIXL1* expression. For endoderm differentiation, cells were treated with 5 uM CHIR in E8 for 24 h and then changed to E8 medium supplemented with activin A (10 ng/ml) for 3 days. Expression of *SOX17* and *FOXA2* was analyzed by RT-qPCR. For ectoderm differentiation, cells were treated with E6 medium (E8 without FGF2 and TGFβ) with 10 μM SB431542 (Selleck) for 1 day and subsequently treated with 10 μM SB431542 and 100 nM LDN193189 (Selleck) in E6 medium for 4 days. Expression levels of *PAX3* and *PAX6* were examined by RT-qPCR.

### Mouse ESC culture and differentiation

Mouse embryonic stem cells (mESCs) were obtained from Deng C.’s laboratory and maintained on MEF using ESF7 medium (1 × DMEM, 15% FBS, 1 × NEAA, 100 µM β-Mercaptoethanol, 1000 U/mL hLIF, 10U/mL Penicillin/Streptomycin) [20]. Medium was changed daily, and cells were passaged using TrypLE/EDTA every 3 days. For trophoblast differentiation, cells were passaged onto gelatin-coated plates in ESF7 medium, and the culture medium was switched the next day to mE5 medium (1 × DMEM/F12, 20 nM sodium selenite, 5 µg/ml holo-transferrin, 10 µg/ml insulin, 10 µM 2-Mercaptoethanol, P/S 10 U/ml Penicillin/Streptomycin) with 20 ng/ml BMP4 for six days with medium change every two days. Cells were harvested on day 6 for analysis.

### Enzyme-linked immunosorbent assay (ELISA)

Human triiodothyronine (T3) ELISA Kit (Herman, YX-200300H) and Human thyroxine (T4) ELISA kit (Herman, YX-200400H) were used to measure the concentration of thyroid hormones. Different medium supplements were diluted in DMEM/F12 to the indicated concentrations, and ELISA was carried out following manufacturer’s instructions. Briefly, samples were added into a 96-well plate precoated with T3 or T4 antibodies, incubated at room temperature for 30 min, and the wells were washed with wash buffer twice. HRP-conjugated secondary antibodies were then added. After incubation and wash, the TMB substrate was added for color development. Stopping buffer was added 15 min later, and the absorbance at 450 nm was measured using a microplate reader. The standard curve was constructed using T3 or T4 standard solutions measured alongside the samples.

### FACS analysis

Cell were dissociated by TrypLE Select (Thermo Fisher, 12563-029) and neutralized by 2 volumes of DMEM/F12 (Thermo Fisher, 11320-033). Cells were collected by centrifugation at 300×*g* for 5 min, washed with PBS, fixed with 4% PFA for 15 min and then washed with PBS. Cells were then permeablized by 0.5% triton X-100 for 15 min and washed with FACS buffer (PBS with 0.05% triton-X100 and 1% BSA). 5 × 10^5^ cells were incubated with primary antibodies for 1 h at room temperature, washed twice with FACS buffer and then incubated with secondary antibodies (Jackson ImmunoResearch Laboratories, Alexa Fluor secondary antibodies) for 1 h. Cells were washed twice with FACS buffer and analyzed by CytoFLEX S flow cytometer (Beckman Coulter). Primary antibodies used include NANOG (R&D, AF1997) and CGB (Abcam, AB9582).

### Cell cycle analysis

Cell cycle analysis was conducted using Click-iT® EdU Flow Cytometry Assay Kits (Thermo Fisher). Briefly, cells were incubated with 10 μM EdU for 30 min and then harvested by TrypLE, and then fixed and permeabilized as described above. EdU was labeled with Alexa Fluor® 647 dye, and DNA was labeled by propidium iodide. Cell cycle profile was analyzed by CytoFLEX S flow cytometer (Beckman Coulter).

### RNA extraction and RT-qPCR analysis

Cell were harvested in RNAiso Plus (TAKARA, 9109) and extracted as instructed by the product manual. 500 ng RNA were added in 10ul reverse transcription reaction mixture using Maxima H Minus Reverse Transcriptase kit (Applied Biosystems, 4368814). The resulting cDNA was diluted 20 times by distilled water, and 2 µl was used for RT-qPCR using SYBR® Premix Ex Taq kit (TAKARA, RR420) on a CFX384 Touch Real-Time PCR Detection System (Biorad). Gene expression was normalized to GAPDH or the control as indicated. Primers for RT-qPCR used in this study are listed in Additional file 5: Table S1.

### Immunostaining

Cells were washed with PBS, fixed with 4% PFA and permeabilized by 0.5% triton-X100. Primary antibodies were applied for 2 h at room temperature. Secondary antibodies (Jackson ImmunoResearch Laboratories, Alexa Fluor® secondary antibodies) were diluted 1:1000 and applied for 1 h at room temperature. All the antibodies were diluted in PBS with 1% BSA. Nuclei were stained with Hoechst 33342, and cell images were captured by EVOS Cell Imaging System (Thermo Fisher). Primary antibodies used include NANOG (R&D, AF1997) and CGB (Abcam, AB9582).

### Western blot analysis

Cells were harvested in 2 × Laemmli buffer (62.5 mM Tris–HCl, pH 6.8, 25% glycerol, 2% SDS, 1 mM NaF, 1 mM Na2P2O4, 1 mM NaVO4, 2.5 nM Glycerol phosphate, 1 mM PMSF). Protein concentrations were determined with BCA protein assay kit (Thermo Fisher Scientific, RE232675). 5 μg total proteins per sample were loaded on SDS-PAGE gel and transferred to PVDF membranes. The membrane was blocked in 5% non-fat-milk in 1 × TBST at room temperature for 1 h. Primary antibodies were applied overnight at 4 °C. Peroxidase-conjugated secondary antibodies (Jackson ImmunoResearch Laboratories, Alexa Fluor® secondary antibodies) were diluted 1:10,000 and applied for 1 h at room temperature. Signals were visualized using SuperSignal™ West Pico Plus Chemiluminescent Substrate Kit (Thermo Fisher, 34577). All the antibodies were diluted in PBS with 1% BSA. Primary antibodies used include GAPDH (Santa Cruz, sc-25778), Phospho-p44/42 MAPK (Erk1/2) antibody (CST, 9101), p44/42 MAPK (Erk1/2) antibody (CST, 9017), phospho-SMAD2 antibody (CST, 3108), Smad2 (D43B4) antibody (CST, 5339).

### Cell apoptosis analysis

To set up high- and low-density culture, H1 hESCs at 70% confluence were passaged by the ratio of 1:3 for high-density culture and 1:12 for low-density culture in E8 or E8 with 500 nM T3, and cultured for 3 days until the high-density culture reached ~ 100% confluence. Cell apoptosis was analyzed by CellEvent™ Caspase-3/7 Green Detection Reagent (Thermo Fisher). Briefly, caspase 3/7 green reagent (2 μM) were added to the cell culture medium and incubated for 30 min at 37 °C. Cells were washed, observed under microscope and then harvested to be analyzed by CytoFLEX S flow cytometer (Beckman Coulter).

### Cell survival after dissociation

High- and low-density H1 culture was set up as described above. Cells from high- or low-density culture were then passaged to new plates using the EDTA method [18] with or without ROCK inhibitor Y-27632 on day 0, and surviving cells were collected using TryPLE Select and cell count determined using CytoFLEX S flow cytometer (Beckman Coulter) on day 1. Survival rate was calculated by day 1 cell count/day 0 cell count.

### Mitochondrial respiration analysis

Mitochondrial respiration was analyzed by Mito Stress Test on a Seahorse XF96 analyzer (Agilent) following published protocols [21]. Prior to the assay, hESCs were cultured in E8 medium with or without T3 for at least 3 days and then passaged to Matrigel-coated XF96 microplate at 2 × 10^4^ cells/well in E8 medium with or without FGF2. Medium was changed the next day with the same treatments added, and Mito Stress Test was run the day after using Agilent Seahorse XF Cell Mito Stress Test Kit, with 500 nM T3 or 100 ng/ml FGF2 added to the assay medium in corresponding wells.

### LC–MS/MS analysis

Quantification of metabolites and data processing were based on previous publications [19, 22]. Briefly, hESCs were cultured in E8 medium with or without T3 for 3 days. For analysis of metabolites in the medium, spent medium was collected and centrifuged at 1000×*g* for 5 min. The supernatant was extracted by nine volumes of extraction solution (75% acetonitrile, 25% methanol, and 0.2% formic acid), vortexed and centrifuged at 10,000 × *g* at 4 °C for 10 min. The supernatant was used for LC–MS/MS analysis. For analysis of intracellular metabolites, cells were washed with 0.9% saline twice, and then, 500 μl/well − 80 °C precooled 80% methanol (containing 10 μg/ml norvaline as internal control) was added. Cells were scraped off and then centrifuged at 12,500×*g* for 15 min. The supernatant was evaporated under nitrogen till dry and redissolved in 100 μl 50% acetonitrile for intracellular metabolic analysis.

Waters Acquity UPLC equipped with Waters Xevo TQD detector was used for LC–MS/MS analysis. The source gas was argon, capillary voltage was 3500 V, and dissolving temperature was 500 °C. Glycolysis and PPP-related metabolites were determined by Acquity UPLC BEH HILIC column (2.1 × 100 mm, 1.7 μm), and other metabolites were determined by Acquity UPLC BEH amide column (2.1 × 100 mm, 1.7 μm). Column temperature was set at 40 °C.

### RNA-seq and bioinformatics analysis

hESCs were treated with different conditions and were then collected with RNAiso Plus (TAKARA, 9109). The RNA sequencing was carried out by Novogene and Genewiz with similar methods. Briefly, total RNA was extracted using RNAiso Plus following manufacturer’s instructions. 1 µg total RNA was used for library preparation. mRNA was extracted using Poly(A) mRNA Magnetic Isolation Module. The sequencing libraries were prepared by KAPA method. Sequencing was performed on the Illumina HiSeq/Novaseq platform, in a 2 × 150 bp paired-end (PE) configuration. The gene reads were aligned to reference genome by Hisat2 (v2.0.1). Reference genome sequences and gene model annotation files were downloaded from ENSEMBL; Hisat2 (v2.0.1) was used to index reference genome sequence; clean data were then aligned to reference genome via software Hisat2 (v2.0.1). The reference genome is homo_GRCh38, and the GTF reference annotation file is in version Homo_sapiens.GRCh38.102. The reads were converted into transcripts by reference transcript annotation. Gene expression levels are calculated from the read numbers that align to exons. The exon-overlapping reads were quantitated using Htseq software following the intersection-non-empty model. Gene read counts were normalized using Transcripts Per Million (TPM). Log2 (TPM of each gene in each sample/mean TPM of each gene in all samples) values were used to generate heatmaps using R package gplots (3.6.1). Differentially expressed genes (DEG) were picked using R package EdgeR based on p value < 0.01, fold change > 1.5 or < − 1.5. The heatmap cluster was established using the Euclidean distances between selected genes in each sample. Gene set enrichment analysis was carried out using Enrichr (https://maayanlab.cloud/Enrichr/).

### Statistical analysis

Data are presented as mean ± SD of three or more biological replicates unless specified. (RT-qPCR results are presented as mean ± SD of two technical repeats.) Statistical analysis was carried out by two-tailed Student’s t test using Excel. *P* < 0.05 was considered statistically significant.

## Results

### Thyroid hormone improves cell survival and passaging efficiency

**Fig. 1:**
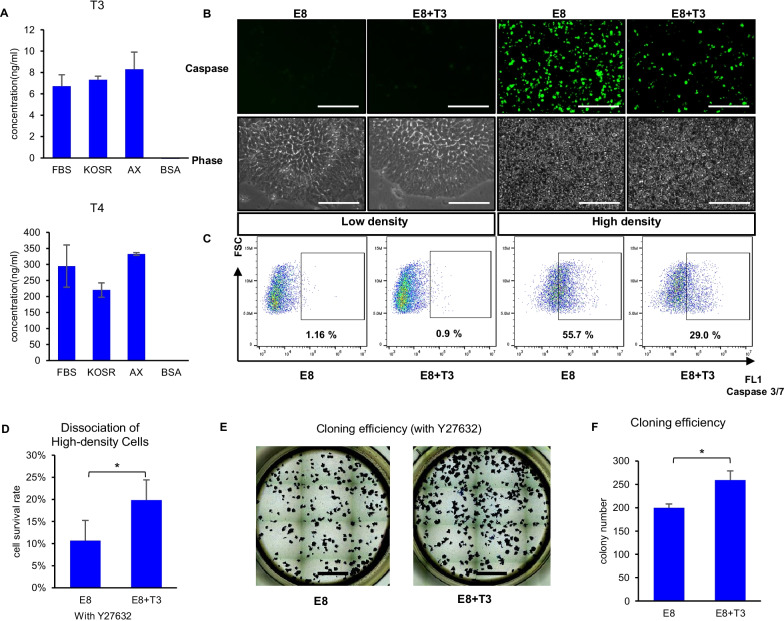
Thyroid hormone T3 improves hPSC high-density survival and cloning efficiency. **A** Concentration of thyroid hormone T3 and T4 in different medium supplements at commonly used concentrations, measured by ELISA. *n* = 3 biological repeats. **B**, **C** Impact of T3 on cell apoptosis in high- and low-density culture treated with or without T3 for 3 days, measured with caspase 3/7 green detection reagent as shown in fluorescence images (**B**) and FACS data (**C**). Scale bar, 100 μm. Data are representative of three independent experiments. **D** T3 enhanced hESC survival after passaging from high-density culture. H1 cells were cultured in E8 or E8 + 500 nM T3 for 3 days until cells reach over 90% confluence, then passaged to a new plate and counted on the next day. *n* = 3 biological repeats, *P < 0.05. **E**, **F** T3 improved cloning efficiency. H1 cells were cultured in E8 or E8 + 500 nM T3 for 3 days and then collected using EDTA for seeding. 500 cells were seeded in each well of a 12-well plate and cultured in E8 or E8 with 500 nM T3 for 6 days. Alkaline phosphatase staining was used to visualize (**E**) and count the colonies (**F**). Scale bar, 5 mm. *n* = 3 biological repeats, *P < 0.05

Given the important roles of thyroid hormone in growth and development and its reported high binding ratio to albumin [17], we examined its presence in traditional culture medium supplements, including AlbuMAX, fetal bovine serum (FBS) and KOSR (Fig. 1A). Both T3 and T4 were present at comparable concentrations in AlbuMAX and KOSR as in FBS, but not in lipid-free bovine serum albumin (BSA). This supports the hypothesis that thyroid hormone could be a factor contributing to the phenotypic differences between feeder/serum/AlbuMAX culture systems and a more defined culture system with or without BSA. We then studied the impact of T3 on hPSCs in chemically defined E8 system.

To examine the role of T3 on hESC survival, we treated H1 cells cultured at high and low cell densities with T3. High cell density (> 90% confluence) leads to medium acidosis and cell death due to the lactic acid released during glycolysis in hESCs [23]. An increase in caspase 3/7-positive cells was observed when cells were cultured at high density (Fig. 1B and 1C), suggesting elevated apoptotic cells. The presence of T3 suppressed cell apoptosis in high-density culture. T3 did not affect cell apoptosis at low cell density. The addition of T3 did not affect the pH of the medium (Additional file 1: Figure S1A), and T3 in combination with bicarbonate (NaHCO_3_) worked synergistically to reduce the percentage of caspase positive cells (Additional file 1: Figure S1B). Their combinatorial effect suggests that T3 improves cell survival through a parallel pathway different from the regulation of acidosis.

Cell dissociation is a necessary step in stem cell passaging, but is also an aggressive procedure which often results in a high cell death rate. Multiple techniques, including the use of ROCK inhibitors during passaging, have been developed, aiming to improve hESC survival after dissociation [24, 25]. High cell density (> 90% confluence) prior to passaging significantly affects cell survival afterward (Fig. 1D and Additional file 1: S1D–F). We examined the effect of T3 on hESC survival after dissociation and found that pretreatment with T3 significantly improved the survival of hESCs from high-density culture (Fig. 1D), even though it cannot replace ROCK inhibition (Additional file 1: Figure S1D). This protective effect was not observed in the passaging of low-density culture, where the pretreatment of cells with T3 did not affect cell survival with or without ROCK inhibitor Y27632 (Additional file 1: Figure S1E and S1F). Transient application of T3 during dissociation did not promote cell survival as T3 pretreatment (Additional file 1: Figure S1G). We also tested if T3 could improve survival when hESCs were plated at cloning density after dissociation and found that T3 significantly improved the cloning efficiency (Fig. 1E and 1F). Taken together, these results suggest that extended treatment with T3 is beneficial for hESC survival.

### Proliferation and pluripotency are maintained in medium with T3

**Fig. 2:**
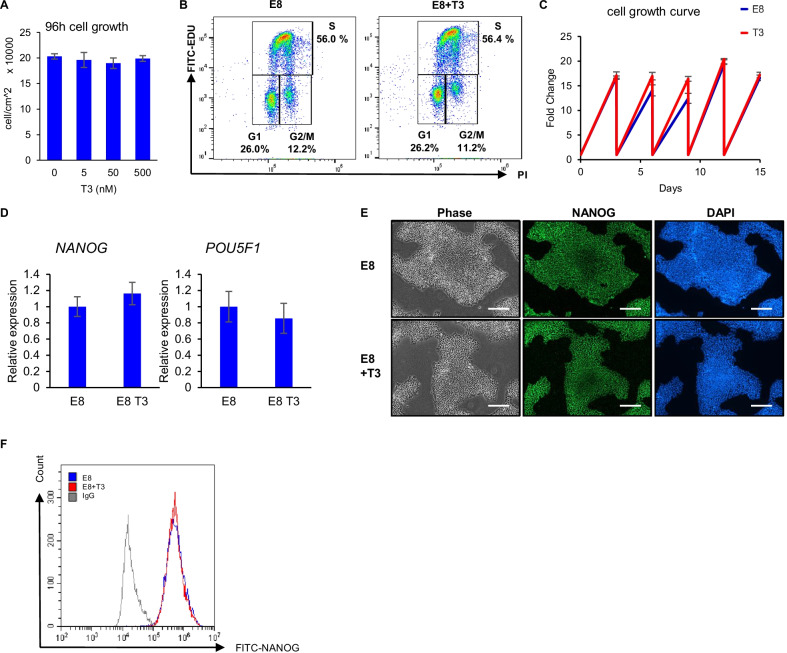
T3 is compatible with long-term hESC maintenance in E8 medium. **A** hESC growth with or without T3. 10,000 H1 cells were seeded in each well of a 24-well plate on day 0 and cultured in E8 or E8 with T3 at different concentrations with daily medium change. Cell count was determined on day 4. *n* = 3 biological repeats. **B** hESC cell cycle profile measured by Click-iT® EdU Flow Cytometry Assay Kit, following pre-treatment with T3 for 3 days. Data are representative of three independent experiments. **C** Continuous expansion of hESCs in the presence of T3. H1 cells were cultured in E8 or E8 with T3 (500 nM) medium in 12-well plates for 5 passages, with 30,000 cells seeded at each passage. Cell counts were determined prior to passaging and normalized to the number seeded. *n* = 3 biological repeats for each time point. **D** Expression of pluripotency markers *NANOG* and *POU5F1* and in H1 cells cultured with or without T3 for five passages, determined by RT-qPCR. GAPDH was used as internal control, and the gene expression was normalized to the level in E8 culture. ns, not significant. Data are representative of three independent experiments. **E** Immunostaining of NANOG in H1 cells cultured with or without T3 for five passages. Scale bar, 200 μm. **F** FACS analysis of NANOG^+^ cells in H1 cells cultured with or without T3 for five passages. Data representative of three independent experiments

We further examined effect of T3 on hESCs under regular maintenance conditions. When T3 was added to E8 medium, a normal hESC growth rate was maintained during a 96-h time period (Fig. 2A). Cell cycle status was not altered significantly by the presence of T3 (Fig. 2B). When hESCs were maintained with T3 for five passages, no significant difference in cell growth was observed compared to normal E8 condition (Fig. 2C). These data suggest that normal growth of hESCs is maintained in the presence of T3.

To study whether T3 treatment affects hESC pluripotency, we examined the expression of pluripotency markers and found that the expression of *NANOG* and *POU5F1* was not altered significantly after treatment with T3 in E8 medium for five passages (Fig. 2D). Immunostaining and flow cytometry analysis showed that > 95% cells were NANOG positive after five passages with T3, similar to regular culture in E8 (Fig. 2E and F). We also demonstrated that normal cell morphology and expression of pluripotency genes were maintained in H9 hESCs and NL-1 hiPSCs (Additional file 2: Figure S2A–D). These data suggest that the addition of T3 in E8 medium for regular cell culture does not alter the growth rate, cell cycle status or pluripotency capacity of hPSCs.

### T3 modulates hESCs metabolism and gene expression

**Fig. 3:**
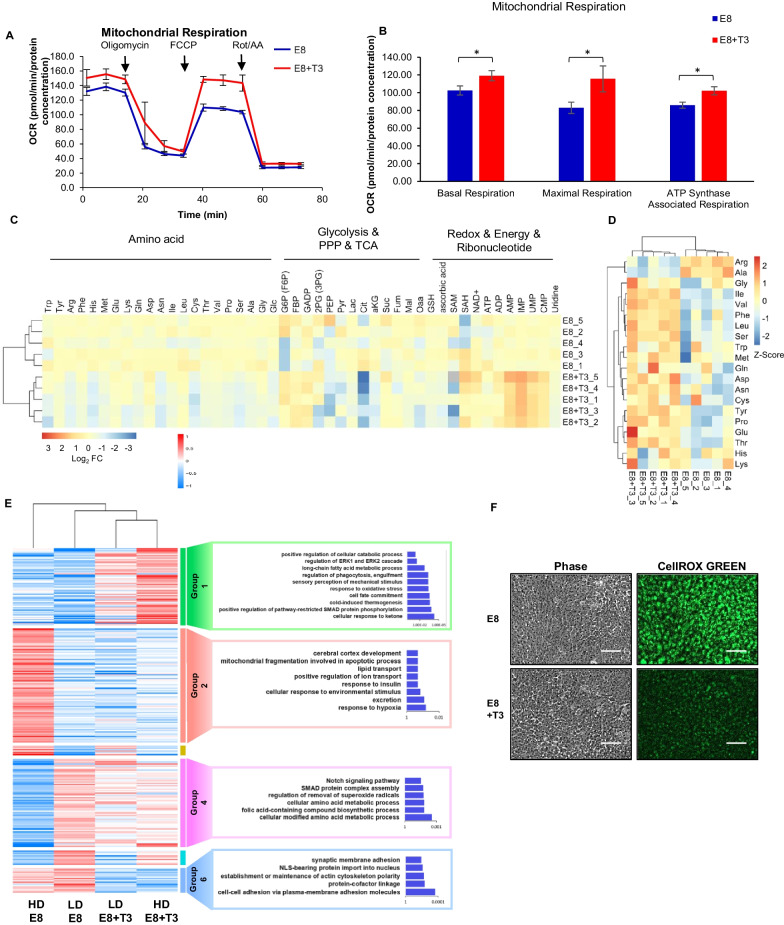
T3 modulates hESC metabolism and gene expression. **A**, **B** T3 modulates hESC mitochondrial respiration. H1 cells treated with E8 or E8 + T3 (500 nM) for 3 days were seeded into the assay plate, cultured for one more day with the same treatment and subjected to the test with T3 added into the assay medium in corresponding wells. Oxygen consumption rate (OCR) of H1 hESCs measured with Mito Stress Test using Seahorse XFe96 analyzer (**A**), basal respiration, maximal respiration and ATP Synthase-Associated Respiration calculated from the assay results in panel A (**B**). *n* ≥ 3, *P < 0.05. **C** Heatmap of intracellular levels of metabolites quantified by LC–MS/MS. Data are shown as log2 fold change of the peak area of corresponding metabolite normalized to the control group (E8). *n* = 5. Red color indicates upregulation and blue downregulation. Gray represents the compound was not detected in the assay. **D** Heatmap of amino acids consumption in culture medium, quantified by LC–MS/MS. Data are shown as Z-Score of the peak area of corresponding metabolite. *n* = 5. Red color indicates more secretion/less consumption, while blue color indicates less secretion/more consumption. **E** RNA-seq analysis of the effect of T3 in high-density (HD, > 90% confluence) and low-density (LD, < 70% confluence) H1 culture. Cells were treated with or without T3 (500 nM) for 3 days. Left, heatmap of RNA-seq DEG. Right, GO term analysis based on clustered genes. **F** T3 reduces ROS level in hESCs. hESC ROS level was detected by CellROX green kit. Scale bar, 100 μm

As T3 is a well-known metabolic regulator [26], we inspected its effect on hESC metabolism. Seahorse XF Cell Mito Stress Test showed that T3 elevated mitochondrial respiration (Fig. 3A and B). T3 significantly upregulated the basal level of oxygen consumption rate (OCR), maximal respiration, ATP synthase-associated respiration as well as spare respiration capacity (Fig. 3B and Additional file 2: S2E). T3 also significantly altered the concentrations of metabolites involved in glycolysis and oxidative phosphorylation (Fig. 3C and Additional file 6: Table S2). Moreover, T3 changed the amino acid profiles in hESCs (Fig. 3D and Additional file 7: Table S3). These results suggest that T3 has profound influence on global metabolism in hESCs.

Both low-density and high-density hESCs treated with T3 were analyzed by RNA-seq (Fig. 3E). T3 upregulated genes involved in lipid metabolism, response to oxidative stress, ERK and SMAD pathways independent of cell density (Group 1). Genes related to cell–cell adhesion, cytoskeleton polarity and protein nuclear translocation were suppressed by T3 treatment (Group 6). Genes related to mitochondrial fragmentation, which is involved in apoptosis, were upregulated by high cell density but suppressed by T3 (Group 2). High cell density also downregulated genes involved in ROS metabolism and translation, such as *DHFRP1*. However, its expression was rescued by T3 (Group 4). It implied that ROS production could be affected by thyroid hormone. We showed that ROS level was indeed suppressed by T3 at high cell density (Fig. 3F). These data suggested that T3 could modulate transcription to influence cellular metabolism.

### T3 promotes pluripotency under suboptimal conditions

**Fig. 4:**
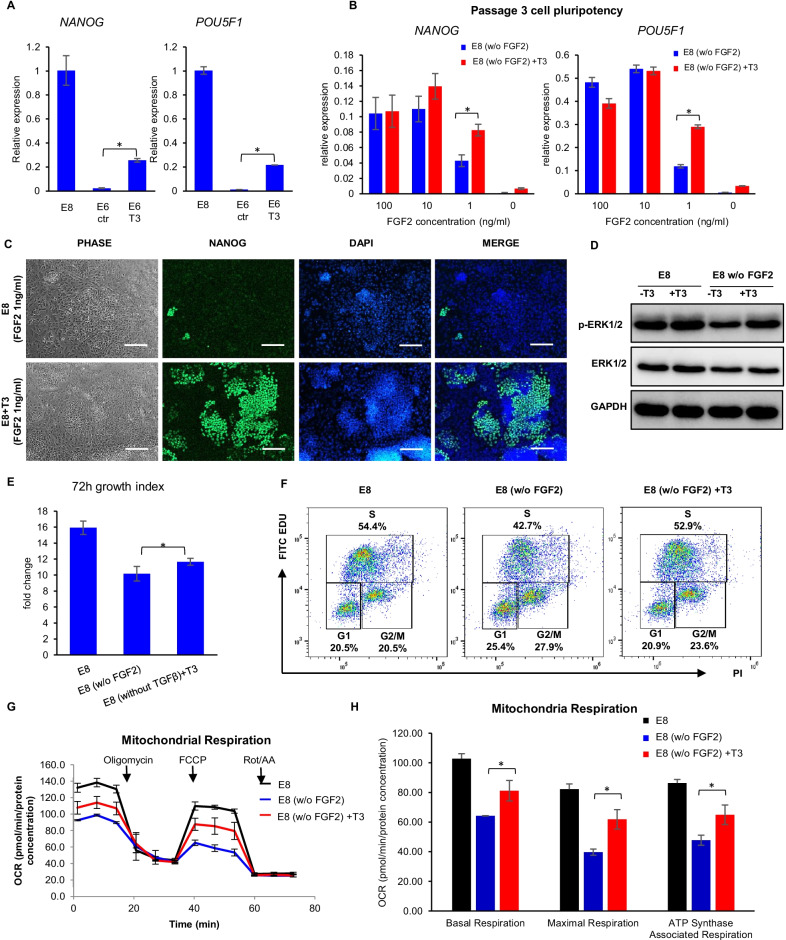
T3 promotes pluripotency under suboptimal conditions. **A** Impact of T3 on spontaneous differentiation. FGF2 and TGFβ were withdrawn from H1 cell culture at 30–40% confluence, and cells were allowed to spontaneously differentiate for 14 days. Medium was changed every two days, and pluripotency markers *NANOG* and *POU5F1* were analyzed by RT-PCR on day 14. GAPDH was used as internal control, and the gene expression were normalized to the levels in E8 culture. Data are representative of three independent experiments. **B** Impact of T3 on pluripotency at low FGF2 concentrations. H1 cells were cultured in E8 medium with different concentrations of FGF2 with or without T3 for 3 passages before analysis by RT-PCR. Gene expression levels were normalized to GAPDH. Data are representative of two independent experiments. **C** Immunostaining of NANOG in H1 cells cultured for 3 passages in E8 medium with 1 ng/ml FGF2 in the presence or absence of T3. Scale bar, 200 μm. **D** Western blot analysis of ERK1/2 (Thr202/Tyr204) phosphorylation. H1 cells were cultured in E8 medium with or without T3 for 4 days with passaging on day 3 and then changed to E8 medium with or without FGF2 for another 24 h before collection for analysis. GAPDH was used as loading control. Data are representative of three independent experiments. **E** T3 promoted hESC growth in the absence of FGF2. H1 cells were cultured with or without FGF2 or T3 (500 nM) for 3 days. *n* = 6 biological repeats. *P < 0.05. **F** Impact of T3 on cell cycle profiles in the absence of FGF2. H1 cells were cultured with or without FGF2 or T3 (500 nM) for 3 days. Data are representative of three independent experiments. **G**, **H** T3 enhances hESC mitochondrial respiration in the medium without FGF2. H1 cells cultured with or without FGF2, the impact of T3 on oxygen consumption rate (OCR) measured by Mito Stress Test (**G**). Basal respiration, maximal respiration and ATP Synthase-Associated Respiration calculated from the assay results in panel **G** (**H**). n ≥ 3, *, P < 0.05

We then examined the impact of T3 on hESC pluripotency in the absence or at low concentrations of growth factors. E8 medium contains FGF2 (100 ng/ml) and TGFβ (1.74 ng/ml) to maintain pluripotency. When FGF2 and TGFβ are removed, hESCs will spontaneously differentiate, and the expression of pluripotency markers will decrease (Fig. 4A). This loss of pluripotency can be partially rescued by T3 (Fig. 4A). When FGF2 is included at lower concentrations in E8 medium for three passages, T3 treatment helped significantly improve *NANOG* and *POU5F1* expression compared to control condition at 1 ng/ml of FGF2 (Fig. 4B and C), suggesting T3 decreases the dependence on FGF2 in defined culture and promotes pluripotency. As FGF2 induces ERK1/2 activation, we examined the level of ERK1/2 phosphorylation and found that T3 elevated ERK1/2 phosphorylation with or without FGF2 (Fig. 4D). This is consistent with the RNA-seq data in which T3 upregulated key genes involved in ERK and SMAD pathways that are essential for pluripotency (Fig. 3E and Additional file 8: Table S4).

Absence of FGF2 from E8 medium leads to decreased cell growth, which may be related to a decreased percentage of S-phase cells (Fig. 4E and F). Both cell growth and cell cycle status are partially rescued by T3 (Fig. 4E and F). In addition, mitochondrial respiration is decreased without FGF2, which is also rescued by T3 (Fig. 4G, H and Additional file 3: S3C). Taken together, these data suggest that T3 decreases the dependence of hPSCs on FGF2.

We also examined the effect of T3 on TGFβ signaling. In the absence of TGFβ, T3 elevated SMAD2 phosphorylation to similar levels found in E8 medium, suggesting that T3 also promotes endogenous TGFβ signaling (Additional file 3: Figure S3A). However, T3 did not rescue cell pluripotency in the absence of TGFβ (Additional file 3: Figure S3B).

### Thyroid hormone promotes trophoblast differentiation

**Fig. 5:**
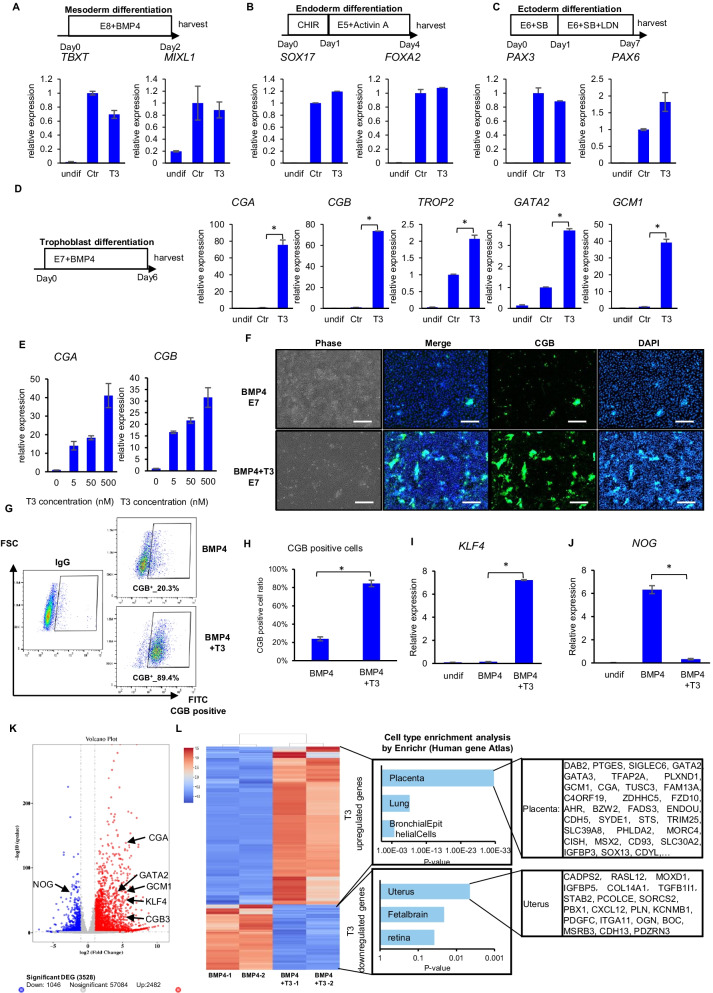
T3 promotes trophoblast differentiation in hESCs. **A**–**D** Effects of T3 on lineage-specific differentiation. Top, schematic drawings of the differentiation protocols. Bottom, analysis of lineage-specific marker genes by RT-qPCR. *TBXT* and *MIXL1* for mesoderm (**A**), *SOX17* and *FOXA2* for endoderm (**B**), *PAX3* and *PAX6* for ectoderm (**C**) and *CGA, CGB, GCM1, GATA2* and *TROP2* for trophoblast (**D**). GAPDH was used as internal reference, and all the gene expression levels were normalized to control (Ctr). Data are representative of three independent experiments, *P < 0.05. E5, E8 medium without TGFβ, FGF2 or insulin. E6, E8 medium without TGFβ or FGF2. E7, E8 medium without FGF2. Undif, undifferentiated cells. **E** Dose-dependent effect of T3 on BMP4-induced trophoblast differentiation. H1 cells were differentiated toward trophoblast linage under BMP4 treatment with different concentrations of T3 for 6 days. Expression of *CGA* and *CGB* was measured by RT-qPCR. GAPDH was used as internal control, and the gene expression levels were normalized to the sample without T3. Data are representative of three independent experiments. **F** Immunostaining showing CGB expression on day 7 of differentiation toward trophoblast lineage with or without T3 (500 nM). Scale bar, 200 µm. **G**, **H** FACS analysis of CGB^+^ cells on day 7 of differentiation. hESCs were induced toward trophoblast lineage by BMP4 with or without T3 (500 nM). *n* = 3 biological repeats, *P < 0.05. **I**, **J** mRNA levels of *KLF4* and *NOG* on day 6 of trophoblast differentiation with or without T3 treatment, measured by RT-qPCR. GAPDH was used as internal control. Data are representative of three independent experiments, *P < 0.05. Undif, undifferentiated cells. **K**, **L** RNA-seq analysis of the effect of T3 in BMP4 induced cell differentiation. hESC was differentiated toward trophoblast under 6 days of BMP4 treatment with or without 500 nM T3. RNA-seq DEGs were visualized by volcano map (**K**), and cell-type enrichment analysis for T3 upregulated and downregulated genes were performed by Enrichr website (**L**)

To examine T3’s effects in lineage-specific differentiation, we used signaling pathway modulators to differentiate hESCs into the three germ layers in the presence or absence of T3. T3 did not significantly affect mesoderm, endoderm or ectoderm differentiation, as shown by the expression of marker genes (Fig. 5A–C). These results confirm that T3 has a positive effect on hESC pluripotency, maintaining the integrity of the inner cell mass inside the blastocyst and allowing for embryo implantation.

We then examined if T3 had any impact on the induction of trophoblast. Trophoblast differentiation is the first cell fate determination process happening in the embryo. The trophoblast and the inner cell mass together form the blastocyst [27, 28]. Since the interaction of trophectoderm and the inner cell mass is essential for the correct implantation and development of the blastocyst, we tested if T3 played a role maintaining this structure. We observed that T3 promoted trophoblast differentiation via BMP4 (Fig. 5D). T3 significantly enhanced the expression of trophoblast genes including *TROP2*, *GATA2*, *CGA*, *CGB* and *GCM1* upon induction with BMP4 in E7 medium (E8 -FGF2) for 6 days (Fig. 5D), and the effect is dose-dependent (Fig. 5E). Similar positive impacts on trophoblast gene expression were also observed in mESCs, H9 hESCs and NL-4 iPSCs (Additional file 4: Figure S4A–C) and were confirmed by immunostaining and FACS analysis (Fig. 5F–H). T3 also upregulated the expression of *KLF4* (Fig. 5I), which is reported to be upregulated in trophoblasts during embryogenesis [29]. BMP pathway antagonist Noggin (encoded by the gene *NOG*) was elevated during trophoblast differentiation, but its expression was significantly suppressed by T3 (Fig. 5J). These data suggested that thyroid hormone might promote trophoblast differentiation by elevating BMP pathway. Finally, RNA-seq analysis was performed to compare the gene expression after BMP4 induced differentiation. T3 significantly elevated the expression of 2482 genes, while suppressed 1046 genes (Fig. 5K). It was consistent with qPCR results that placenta marker genes such as CGA and CGB were elevated by T3, and KLF4 and Noggin were differentially affected. Cell-type enrichment analysis shows T3 promoted cell types enriched in placenta (Fig. 5L), while suppressed mesoderm and ectoderm differentiation related to uterus and brain (Fig. 5M).

## Discussion

Pluripotent stem cells hold great promises in regenerative medicine, but their applications are limited by the quality of cell culture and composition of stem cell culture systems. Thyroid hormones are essential for human metabolism, growth and development, but their function in stem cell applications has rarely been discussed. In this report, we demonstrate that thyroid hormone T3 improves hPSC maintenance, enhances cell survival in high density and reduces the need for high concentration of growth factors. T3 also promotes trophoblast differentiation under BMP4 induction.

Although thyroid hormone is reported to be critical for in vitro fertilization and embryogenesis, its molecular mechanism at blastocyst stage is unclear. Here, we demonstrate that T3 promotes trophoblast differentiation under BMP4 induction by suppressing BMP antagonist Noggin. On the other hand, T3 helps maintain hESC pluripotency under suboptimal conditions during continuous cell expansion, not interfering with lineage specification. These results suggest that T3 benefits both blastocyst cell types, trophoblast and inner cell mass.

Thyroid hormone is well known for its transcriptional regulation through nuclear receptors. In this study, T3 treatment altered the expression of genes involved in metabolism and oxidative stress and downregulated proapoptotic genes at high cell density, consistent with its role as a transcription regulator. In addition, T3 promoted oxidative phosphorylation as shown by extracellular flux analysis. Although glycolysis has been known as the major glucometabolic process in hESCs, it is also reported that mitochondrial respiration is of key importance in the survival of iPSCs [30]. In a previous study, we showed that the insulin–AKT pathway promotes mitochondrial respiration to improve hESC survival [31]. Based on our results, it is plausible that T3 may also improve hPSC survival by maintaining mitochondrial respiration.

In this study, we showed that T3 suppressed the expression of BMP antagonist Noggin during trophoblast differentiation. It is consistent with T3’s role during chondrocyte differentiation, when T3 promotes BMP4 production but suppresses Noggin [32, 33]. We also demonstrated that T3 upregulated key transcriptional factors, such as *KLF4* and *KLF9* in hESCs (Additional file 8: Table S4), and also elevated *KLF4* expression during trophoblast differentiation (Fig. 5I, K). T3 was reported regulates oligodendrocyte and hepatocyte differentiation through thyroid hormone/*KLF9* axis [34, 35] and is also frequently used during maturation process of specific cell types, such as pancreatic islets, kidney and muscle stem cells [36–38]. It would be interesting to examine whether T3 regulates differentiation and maturation through similar pathways.

Distinct hPSC phenotypes have been observed in chemically defined E8 medium and AlbuMAX-containing feeder cell culture. In comparison with defined medium, feeder cell culture requires significantly less FGF2 to maintain pluripotency, and trophoblast linage could be efficiently induced by BMP4 in AlbuMAX-containing culture systems [39]. We show that thyroid hormones, including T3 and T4, are present in AlbuMAX. Addition of T3 into defined medium helped maintain pluripotency at low concentration of FGF2 and significantly promoted trophoblast differentiation in defined culture. Thyroid hormones probably played significant roles in regard to the apparent differences between the two culture systems. Because AlbuMAX also contains other functional factors such as albumin and lipids, it would be interesting to study how they could influence hPSCs jointly with thyroid hormones.

## Conclusions

In summary, we identified thyroid hormone as a bioactive component in feeder cell culture and demonstrated its beneficial effects on hESC maintenance and differentiation in chemically defined medium. T3 enhances cell survival and pluripotency during hPSC maintenance by modulating gene expression and metabolism and promotes trophoblast induction by BMP4 through KLF4 and Noggin. Thyroid hormone could serve as an important tool to improve the consistency of stem cell culture and improve lineage-specific differentiation.

## Supplementary Information

**Additional file 1: Figure S1**. Thyroid hormone T3 improves hPSC high-density survival and cloning efficiency. Related to Figure 1. A. T3 does not affect medium pH after 24h of culture. H1 cells were cultured at low density (<70% confluence) or high density (>90% confluence) with or without 500 nM T3. Medium pH was measured using a pH meter after 24 hours. n = 3 biological repeats. B. Immunostaining showing effects of T3 and NaHCO3 on caspase 3/7 activity in high-density H1 culture. H1 cells were cultured in E8 with or without NaHCO3 (20mM) and T3 (500nM) for 3 days until 100% confluence. Caspase 3/7 activity were detected using caspase 3/7 green detection reagent. Scale bar, 100 μm. C. FACS analysis of caspase 3/7 activity in high-density H1 culture. H1 cells were cultured in E8 with or without NaHCO3 (20mM) and T3 (500nM) for 3 days until 100% confluence. Caspase 3/7 activity were detected using caspase 3/7 green detection reagent. D-F. Impact of T3 on hESC survival after passaging from high- or low-density culture. H1 cells were pretreated with or without 500 nM T3 at high or low density, then passaged to a new plate with or without ROCK inhibitor Y27632 and counted on the next day. G. Impact of transient application of T3 during hESC passaging from high-density culture. H1 cells were cultured in E8 medium until high density without pretreatment, then passaged to a new plate in the presence or absence of T3 (500nM) and counted on the next day

**Additional file 2: Figure S2**. T3 is compatible with long-term hESC maintenance in E8 medium. Related to Figure 2. A. Effect of T3 on pluripotency in H9 hESCs. H9 cells were maintained in E8 medium with or without T3 (500nM) for five passages, and the expression of pluripotency markers *NANOG* and *POU5F1* were examined by real time PCR. GAPDH was used as internal control and gene expression was normalized to the level in E8 culture. B. Morphology of H9 hESCs cultured with or without T3 for five passages. Scale bar, 200µm. C. Effect of T3 on pluripotency in NL-1 hiPSC. NL-1 cells were maintained in E8 medium with or without T3 (500nM) for five passages, and the expression of pluripotency markers *NANOG* and *POU5F1* were examined by real time PCR. GAPDH was used as internal control and gene expression was normalized to the level in E8 culture. D. Morphology of NL-1 hiPSCs cultured with or without T3 for five passages. Scale bar, 200µm. E. T3 enhances hESC mitochondrial spare respiration capacity

**Additional file 3: Figure S3**. T3 promotes pluripotency under suboptimal conditions. Related to Figure 4. A. Western blot analysis of Smad2 (Ser245/250/255) phosphorylation. H1 cells were cultured in E8 medium with or without T3 for 4 days with passaging on day 3, and then changed to E8 medium with or without TGFβ for another 24 hours before collection for analysis. GAPDH was used as loading control. Data are representative of three independent experiments. B. Effect of T3 on pluripotency in the absence of TGFβ. H1 cells were cultured in E8 medium without TGFβ for 5 passages in the presence or absence of T3, and the mRNA levels of pluripotency marker *NANOG* and *POU5F1* were analyzed by qPCR. GAPDH was used as internal control, and gene expression was normalized to the level in E8 culture. Data are representative of three independent experiments. C. T3 enhances hESC mitochondrial spare respiration capacity in the medium without FGF2

**Additional file 4: Figure S4**. T3 promotes trophoblast differentiation in hESCs. Related to Figure 5. A. Effect of T3 on BMP4-induced trophoblast differentiation in mESCs. mESC were differentiated toward trophoblast linage under BMP4 treatment in the absence or presence of 500 nM T3 for 6 days. The expression of *Gata2*, *Gata3* and *Hand1* were analyzed by real time PCR. B-C. Effect of T3 on BMP4-induced trophoblast differentiation in H9 hESCs (B) and NL-1 hiPSCs (C). H9 and NL-1 cells were differentiated toward trophoblast linage under BMP4 treatment in the absence or presence of 500 nM T3 for 6 days. The expression of *CGA*, *CGB*, *GCM1* and *GATA2* were analyzed by real time PCR

**Additional file 5**. **Table S1.** Primers used for real time PCR.

**Additional file 6**. **Table S2.** LC-MS/MS analysis of intracellular levels of metabolites.

**Additional file 7**. **Table S3.** LC-MS/MS analysis of amino acid consumption in culture medium.

**Additional file 8**. **Table S4.** GO term of the RNA-seq analysis.

## Data Availability

The datasets used and/or analyzed during the current study are available from the corresponding author on reasonable request.

## Abbreviations

T3: Triiodothyronine
T4: Thyroxine
hPSCs: Human pluripotent stem cells
hESCs: Human embryonic stem cells
mESCs: Mouse embryonic stem cells
FACS: Fluorescence-activated cell sorting
LC–MS: Liquid chromatography–mass spectrometry
RNA-seq: Ribonucleic acid sequencing
KOSR: Knockout Serum Replacement
IVF: In vitro fertilization
hCG: Human chorionic gonadotropin
FBS: Fetal bovine serum
BSA: Bovine serum albumin
hLIF: Human leukemia inhibitory factor
ELISA: Enzyme-linked immunosorbent assay
HRP: Horseradish peroxidase
TMB: 3,3′,5,5′-Tetramethylbenzidine
PFA: Paraformaldehyde
ROS: Reactive oxygen species
