# Supplementary figures and images for: Thyroid hormone enhances stem cell maintenance and promotes lineage-specific differentiation in human embryonic stem cells
Source: Stem Cell Res Ther. 2022 Mar 21;13:120. doi: 10.1186/s13287-022-02799-y (PMC8935725; doi:10.1186/s13287-022-02799-y)

Figure S1

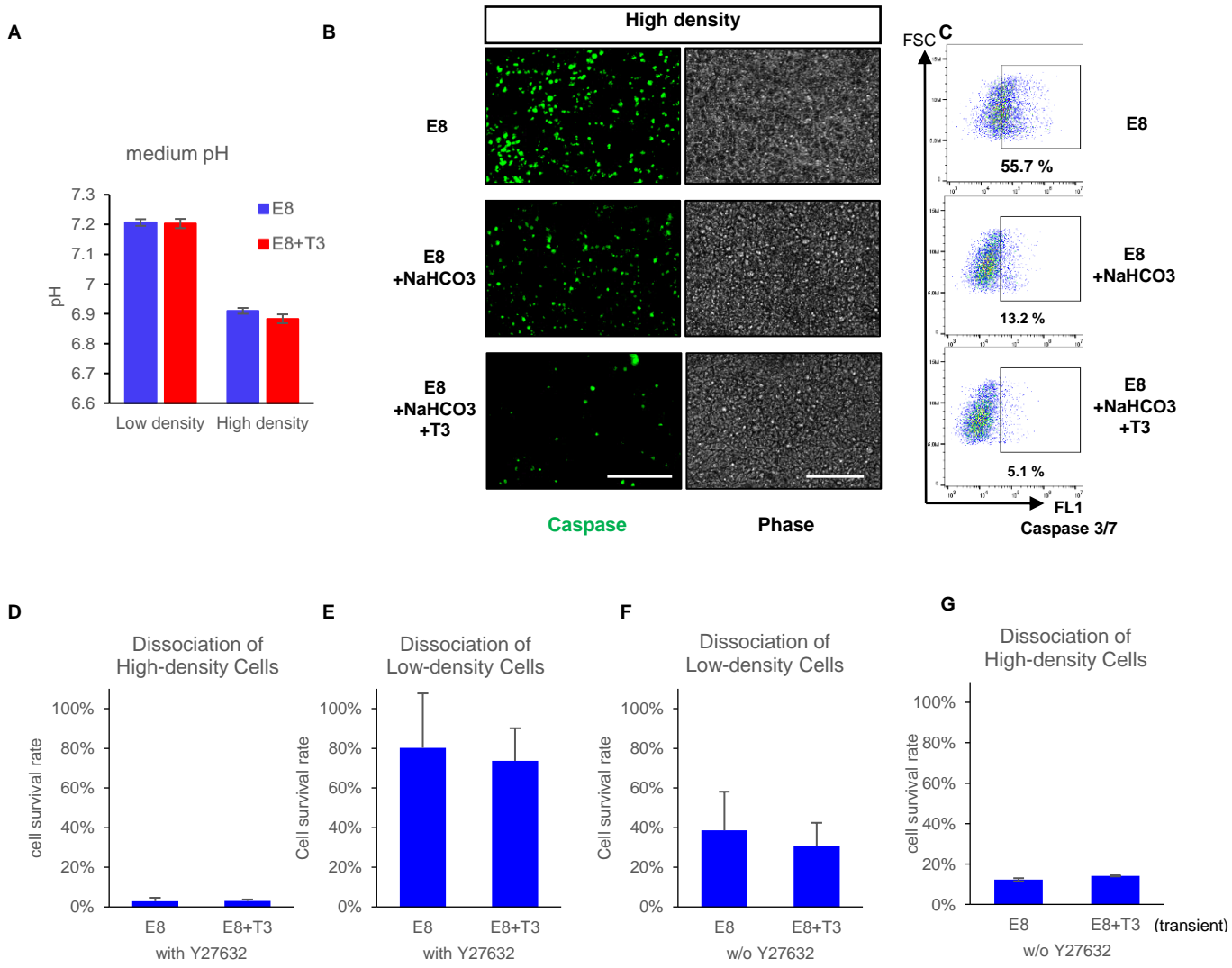

Supplement: Supplementary file 1 — Additional file 1: Figure S1. Thyroid hormone T3 improves hPSC high-density survival and cloning efficiency. Related to Figure 1. A. T3 does not affect medium pH after 24h of culture. H1 cells were cultured at low density (<70% confluence) or high density (>90% confluence) with or without 500 nM T3. Medium pH was measured using a pH meter after 24 hours. n = 3 biological repeats. B. Immunostaining showing effects of T3 and NaHCO3 on caspase 3/7 activity in high-density H1 culture. H1 cells were cultured in E8 with or without NaHCO3 (20mM) and T3 (500nM) for 3 days until 100% confluence. Caspase 3/7 activity were detected using caspase 3/7 green detection reagent. Scale bar, 100 μm. C. FACS analysis of caspase 3/7 activity in high-density H1 culture. H1 cells were cultured in E8 with or without NaHCO3 (20mM) and T3 (500nM) for 3 days until 100% confluence. Caspase 3/7 activity were detected using caspase 3/7 green detection reagent. D-F. Impact of T3 on hESC survival after passaging from high- or low-density culture. H1 cells were pretreated with or without 500 nM T3 at high or low density, then passaged to a new plate with or without ROCK inhibitor Y27632 and counted on the next day. G. Impact of transient application of T3 during hESC passaging from high-density culture. H1 cells were cultured in E8 medium until high density without pretreatment, then passaged to a new plate in the presence or absence of T3 (500nM) and counted on the next day [file 13287_2022_2799_MOESM1_ESM.pdf]

Figure S2

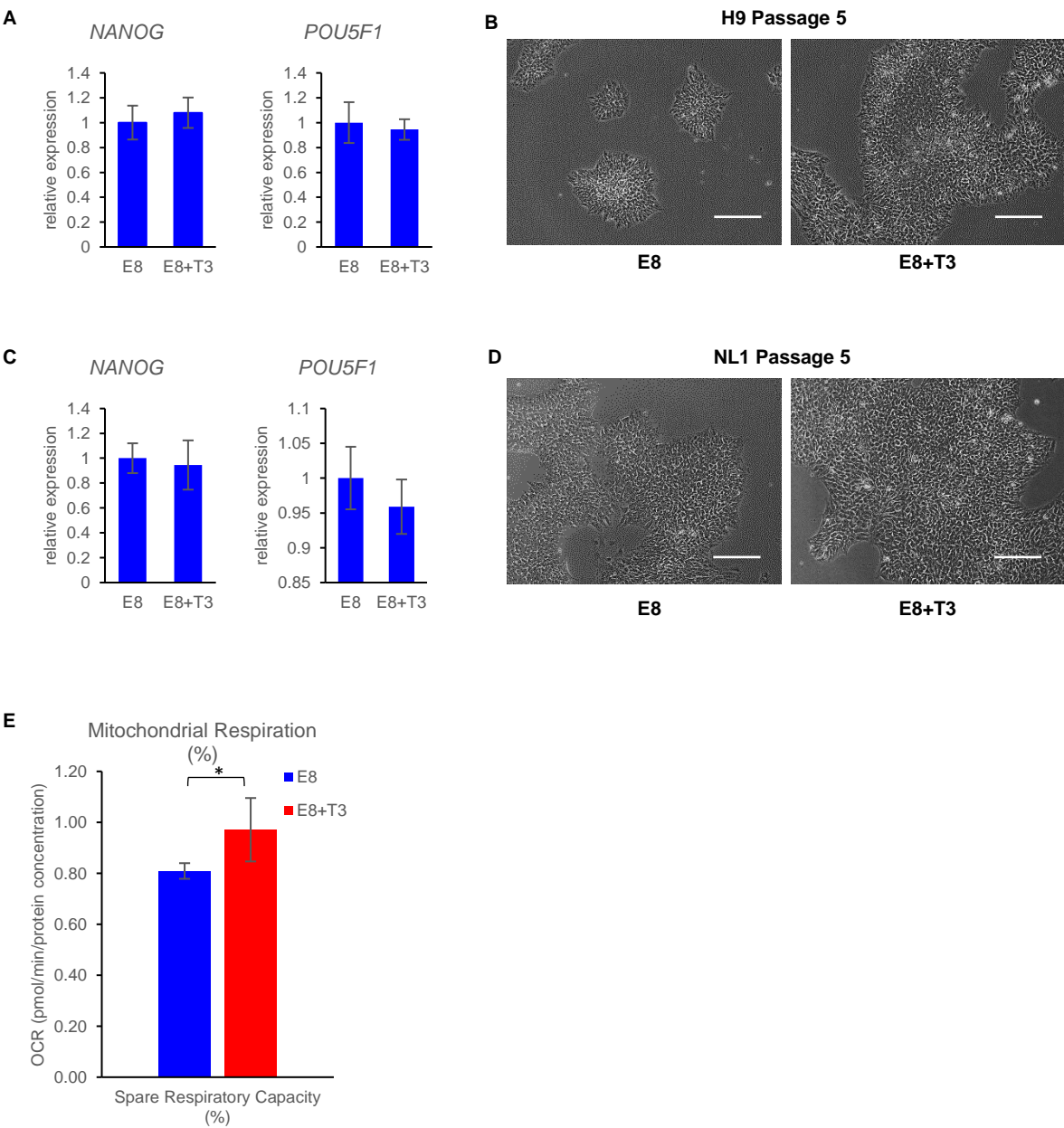

Supplement: Supplementary file 2 — Additional file 2: Figure S2. T3 is compatible with long-term hESC maintenance in E8 medium. Related to Figure 2. A. Effect of T3 on pluripotency in H9 hESCs. H9 cells were maintained in E8 medium with or without T3 (500nM) for five passages, and the expression of pluripotency markers NANOG and POU5F1 were examined by real time PCR. GAPDH was used as internal control and gene expression was normalized to the level in E8 culture. B. Morphology of H9 hESCs cultured with or without T3 for five passages. Scale bar, 200µm. C. Effect of T3 on pluripotency in NL-1 hiPSC. NL-1 cells were maintained in E8 medium with or without T3 (500nM) for five passages, and the expression of pluripotency markers NANOG and POU5F1 were examined by real time PCR. GAPDH was used as internal control and gene expression was normalized to the level in E8 culture. D. Morphology of NL-1 hiPSCs cultured with or without T3 for five passages. Scale bar, 200µm. E. T3 enhances hESC mitochondrial spare respiration capacity [file 13287_2022_2799_MOESM2_ESM.pdf]

Figure S3

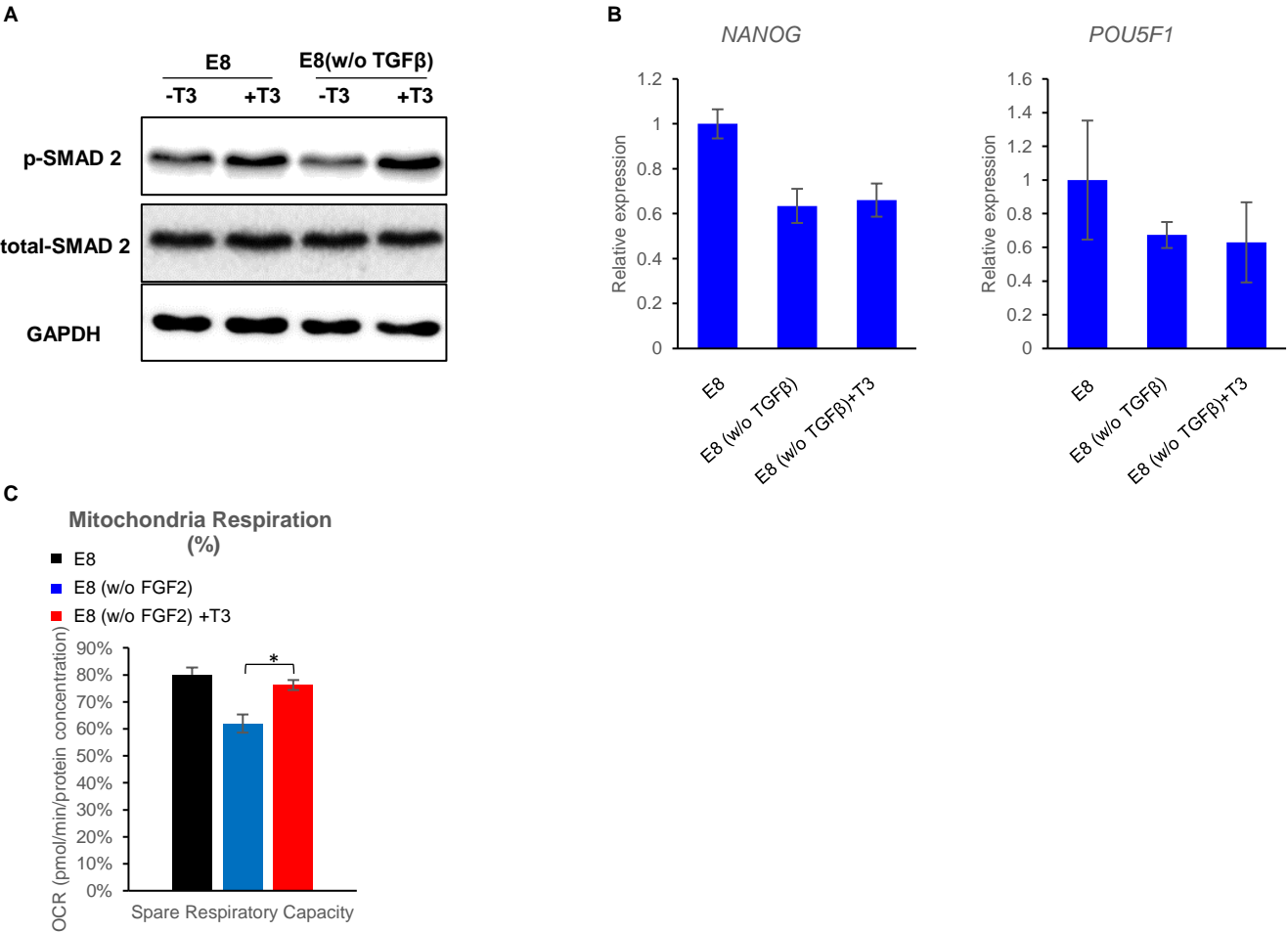

Supplement: Supplementary file 3 — Additional file 3: Figure S3. T3 promotes pluripotency under suboptimal conditions. Related to Figure 4. A. Western blot analysis of Smad2 (Ser245/250/255) phosphorylation. H1 cells were cultured in E8 medium with or without T3 for 4 days with passaging on day 3, and then changed to E8 medium with or without TGFβ for another 24 hours before collection for analysis. GAPDH was used as loading control. Data are representative of three independent experiments. B. Effect of T3 on pluripotency in the absence of TGFβ. H1 cells were cultured in E8 medium without TGFβ for 5 passages in the presence or absence of T3, and the mRNA levels of pluripotency marker NANOG and POU5F1 were analyzed by qPCR. GAPDH was used as internal control, and gene expression was normalized to the level in E8 culture. Data are representative of three independent experiments. C. T3 enhances hESC mitochondrial spare respiration capacity in the medium without FGF2 [file 13287_2022_2799_MOESM3_ESM.pdf]

Figure S4

A

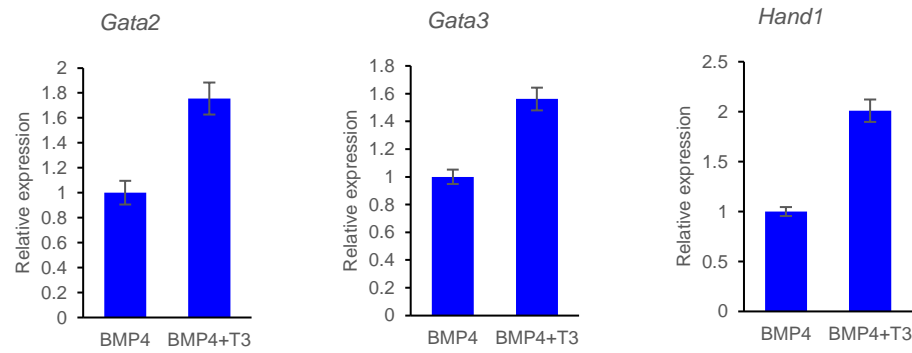

B

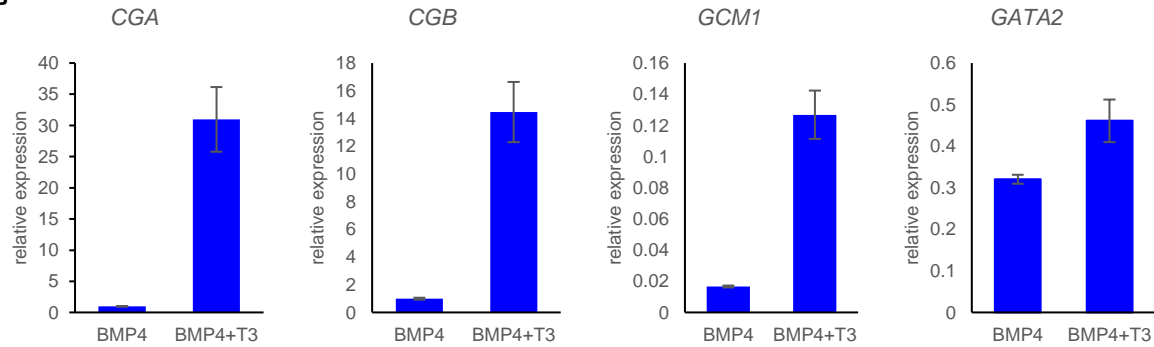

C

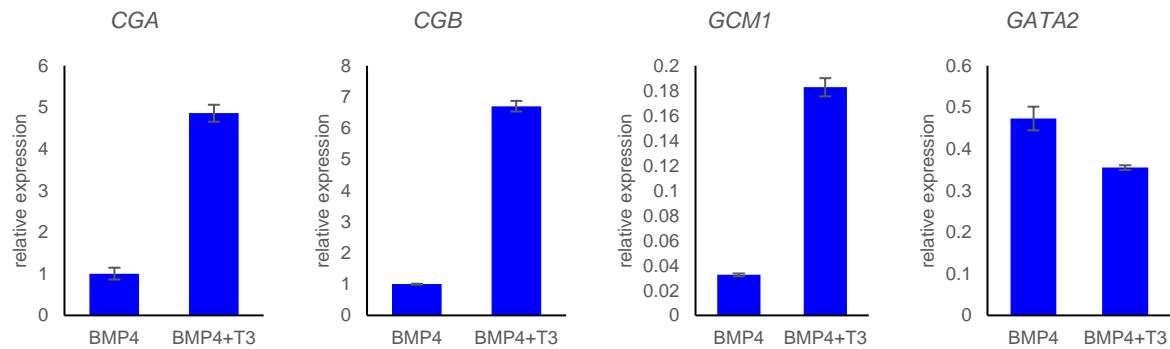

Supplement: Supplementary file 4 — Additional file 4: Figure S4. T3 promotes trophoblast differentiation in hESCs. Related to Figure 5. A. Effect of T3 on BMP4-induced trophoblast differentiation in mESCs. mESC were differentiated toward trophoblast linage under BMP4 treatment in the absence or presence of 500 nM T3 for 6 days. The expression of Gata2, Gata3 and Hand1 were analyzed by real time PCR. B-C. Effect of T3 on BMP4-induced trophoblast differentiation in H9 hESCs (B) and NL-1 hiPSCs (C). H9 and NL-1 cells were differentiated toward trophoblast linage under BMP4 treatment in the absence or presence of 500 nM T3 for 6 days. The expression of CGA, CGB, GCM1 and GATA2 were analyzed by real time PCR [file 13287_2022_2799_MOESM4_ESM.pdf]
